# An Observational Case-Control Study of the Anti-Inflammatory Effects of Bromelain on Peripheral Blood Mononuclear Cells from Patients with Systemic Lupus Erythematosus

**DOI:** 10.31138/mjr.111125.air

**Published:** 2026-06-01

**Authors:** Abhijit Pati, Bidyut K. Das, Sarit S. Pattanaik, Aditya K. Panda

**Affiliations:** 1 ImmGen EvSys Lab, BT-113, Department of Biotechnology, Berhampur University, Bhanja Bihar, Berhampur, Odisha, India;; 2 Department of Clinical Immunology and Rheumatology, SCB Medical College, Cuttack, Odisha, India; 3 Centre of Excellence on Bioprospecting of Ethno-pharmaceuticals of Southern Odisha (CoE-BESO), Berhampur University, Berhampur, India

**Keywords:** bromelains, peripheral blood mononuclear cells, cytokines, inflammation, Systemic Lupus Erythematosus

## Abstract

**Background:**

Bromelain is a proteolytic enzyme that has been investigated as an adjuvant for treating autoimmune disorders. However, data regarding the role of bromelain in systemic lupus erythematosus is limited. In the present study, we examined the effect of bromelain on the mononuclear cells of SLE patients, precisely its anti-inflammatory properties.

**Materials and Methods:**

Peripheral blood mononuclear cells (PBMCs) from SLE patients and healthy controls were stimulated with bromelain (10μg/ml), lipopolysaccharide (10μg/ml), or without any stimulant and cultured for 12 hours. The supernatants were harvested and quantified for IL-1β, TNF-α, IL-6, and IL-17A levels. The mean cytokine levels were compared among different groups using ANOVA and Tukey’s post hoc test.

**Results:**

Our study revealed that PBMCs of SLE patients (n=41) had significantly higher levels of IL-6 (P=0.0003) and IL-1β (P<0.0001) compared to healthy controls (n=36). When stimulated with lipopolysaccharide, PBMCs of SLE patients showed even higher cytokine levels than healthy controls (IL-6:P<0.0001, TNF-α:P=0.01; IL-1β:P<0.0001). However, the most significant finding was that PBMCs stimulated with bromelain exhibited reduced levels of IL-1β (P<0.0001) and TNF-α (P<0.0001) compared to unstimulated cells of SLE patients. Moreover, cells subjected to lipopolysaccharide and bromelain stimulation displayed lower levels of all cytokines than those stimulated with lipopolysaccharide alone (IL-6:P=0.005, TNF-α:P<0.0001, IL-1β:P<0.0001, IL-17A:P=0.02).

**Conclusions:**

The present study highlights the anti-inflammatory role of bromelain in SLE patients by reducing the production of pro-inflammatory cytokine levels in vitro cultured PBMCs derived from SLE patients. Bromelain may be an efficacious adjuvant in managing inflammation in SLE; however, further studies are required.

## INTRODUCTION

Systemic lupus erythematosus (SLE) is a chronic autoimmune disease characterised by the production of autoantibodies and a wide range of clinical manifestations such as skin rashes, joint pain, kidney dysfunction, and other organ involvement.^1–3^ Cytokines play an important role in the development and progression of SLE as higher inflammatory conditions and immune modulation characterise the autoimmune disease. Overproduction of pro-inflammatory cytokines, such as tumour necrosis factor-alpha (TNF-α) and type I interferons (IFN-α), have been reported in patients, linked with various tissue damage.^4–7^ In response to the autoantigens, the activated immune cells, including plasmacytoid dendritic cells, release IFN-α.^8^ In addition, the Interleukin-6 (IL-6) stimulates B-cell differentiation into antibody-secreting plasma cells, which further exacerbates the inflammation in SLE.^9–14^ Cytokine dysregulation plays a crucial role in sustaining inflammatory processes and contributes to the formation of immune complexes, which are fundamental to the pathophysiology of SLE. This dysregulation instigates localised inflammation and subsequent tissue damage, particularly affecting renal structures and leading to the development of lupus nephritis.^15^ Deposition of immune complexes activates complement pathways and recruits inflammatory cells, resulting in the release of additional cytokines that sustain the inflammatory cycle.^16^ This hyper-inflammatory condition features excessive activation of T lymphocytes and macrophages, leading to an overproduction of cytokines and systemic effects of the disease.^17^ Chronic inflammation in SLE is closely linked to an elevated risk of cardiovascular disease^18^ and various comorbidities. This relationship highlights the critical importance of implementing effective strategies for the management of inflammation in patients with SLE.^19^

SLE is managed through long-term immunosuppressive therapies, which suppress the immune system, increasing the risk of infections, malignancies, and organ toxicity.^20,21^ Conventional treatments, including corticosteroids, cyclophosphamide, and mycophenolate mofetil, show efficacy but cause adverse effects such as osteoporosis, diabetes, liver dysfunction, and cardiovascular complications.^20,21^ These effects necessitate developing safer, targeted therapeutic approaches that control the disease without compromising immune function. Emerging biological agents and precision medicine strategies show promise, but further research is needed to optimise efficacy and safety.

Bromelain, an enzyme from pineapple (Ananas comosus), has potent anti-inflammatory properties.^22^ Research shows bromelain inhibits inflammatory mediators, including prostaglandin E2 and thromboxane A2, which are crucial in inflammation.^23^ Furthermore, bromelain reduces pro-inflammatory cytokines IL-1β, IL-6, and TNF-α, and the transcription factor NF-кB, which regulates immune responses.^24^ This multi-targeted mechanism underscores bromelain’s potential as a complementary therapeutic agent for chronic inflammation.

Clinical studies have shown bromelain’s efficacy in reducing pain and inflammation across medical contexts. Acharya et al.^25^ compared bromelain and other enzymes to NSAIDs in patients with fractures, finding bromelain effective in alleviating pain and inflammation while reducing NSAID-associated side effects. This suggests bromelain’s potential as an alternative or adjunct to traditional anti-inflammatory medications. SLE is characterised by elevated levels of inflammatory molecules, and one potential approach to managing SLE involves reducing these inflammatory molecules. In the present investigation, we hypothesised that bromelain may influence disease mechanisms in vitro by reducing inflammatory markers. The present study aims to investigate bromelain’s impact on immune cells isolated from SLE patients through an in-vitro model, based on the hypothesis that bromelain can mitigate inflammation by modulating immune responses. An in-vitro study is essential as it provides a controlled environment to directly examine bromelain’s effects on immune cell function, cytokine production, and inflammation pathways in SLE. These preliminary data will be crucial in determining bromelain’s therapeutic potential and could inform future clinical trials.

## MATERIALS AND METHODS

### Study Participants

The inclusion and exclusion criteria were established prior to the commencement of the investigation. The inclusion criteria for this study encompass female patients diagnosed with SLE according to the American College of Rheumatology criteria (Harley et al., 2009), who exhibit active disease with a disease activity score exceeding four and have provided informed consent. Additionally, healthy female controls without autoimmune disorders are included for comparative purposes. The exclusion criteria comprise conditions that may interfere with study outcomes or elevate risk, such as the presence of other auto-immune diseases, recent infections, significant comorbidities, the use of immunosuppressants beyond standard treatments, pregnancy or lactation, and the inability to consent or adhere to study protocols. The current study involved female patients with SLE who either visited or were hospitalised in the Department of Rheumatology at SCB Medical College. All patients were evaluated clinically by trained clinicians. In addition, standard biochemical tests were performed on all patients to ensure appropriate clinical categorisation. Details of baseline characteristics and treatment strategies are shown in **Table 1**. Healthy females without a history of autoimmune disorders were considered as controls. About 5 mL of intravenous blood was collected in anticoagulant vials from all participants. The Institutional Ethical Committee of SCB Medical College, approved the study protocol (IEC/623/10.03.2021), and written informed consent was obtained from each subject.

**Table 1.** Baseline characteristics of subjects enrolled in the present study.

**Clinical profiles**	**SLE (n = 41)**	**HC (n =36)**
Sex, male/female	0/41	0/36
Age, years, mean ± SD	27.65±7.84	28.01±8.36
Duration of disease, years, mean ± SD	2.83 ± 2.24	-
Disease onset, years, mean ± SD	26.46 ± 7.97	-
SLEDAI scores, mean ± SD	10.26 ± 6.34	-
*ACR criteria*		
Photosensitivity	11 (28)	-
Malar rash	18 (43)	-
Discoid rash	9 (22)	-
Oral ulcer	24 (58)	-
Arthritis	21 (52)	-
NPSLE	10 (25)	-
Carditis	6 (14)	-
AIHA	3 (8)	-
Serositis	5 (12)	-
Nephritis	22 (54)	-
Pneumonitis	3 (8)	-
*Treatments details*		
Prednisolone (5 – 50 mg)	41 (100)	-
Hydroxychloroquine, 6.5 mg/kg body weight	41 (100)	-
Calcium, 1g / day	41 (100)	-
Vitamin D_3_, 250 – 500 IU/day	41 (100)	-
Azathioprine, 50 – 100 mg/day	4 (10)	-
Mycophenolate mofetil, 2gm/day	3 (7)	-

Data are shown in numbers (%) unless otherwise specified. HC: healthy controls; SLE: systemic lupus erythematosus; SD: standard deviation; ACR: American College of Rheumatology; NPSLE: neuropsychiatric systemic lupus erythematosus; AIHA: autoimmune haemolytic anaemia.

### Cell Isolation and Culture

Collected blood samples were centrifuged at 1500g for 10 minutes. The supernatant was carefully transferred to a new sterile tube using a pipette, avoiding the buffy coat and red blood cell layers for subsequent use as autologous plasma. Approximately 3 mL of sterile phosphate-buffered saline (PBS) was added to the pellet. Peripheral blood mononuclear cells (PBMCs) were isolated using density gradient centrifugation with HiSepTM LSM 1077 (HiMedia). Approximately 4 mL of HiSepTM 1077 was placed in a 10 mL centrifuge tube, and the diluted blood was carefully layered over it and centrifuged at 400 x g for 30 minutes at room temperature without brake. The PBMC layer was carefully collected by pipette, washed twice with RPMI 1640 media, and centrifuged at 200 x g for 10 minutes to remove platelets. The cell pellet was resuspended in RPMI-1640 media, counted using a haemocytometer, and adjusted to a concentration of 1 x 10^6^ cells/mL in the media supplemented with 1% of antibiotic solution (Hi-Media A014) and 10% autologous plasma. Cells were cultured in 6-well plates at 37°C in a humidified incubator with 5% CO_2_ for six hours. The non-adherent cells were removed by washing, and the adherent cells were stimulated with appropriate molecules and incubated for 12 hours. After 12 hours of incubation, the supernatants were harvested and analysed for cytokine levels.

### Cell viability test

To determine the optimal levels of bromelain and lipopolysaccharide for their effects and to minimise cell death rate, cells subjected to titration of different doses of LPS and bromelain were harvested using a cell scraper after the cultured supernatants were removed from the culture plates. The isolated cells were stained with trypan blue and assessed for cell viability following 12 hours of culture by a haemocytometer.

### Cytokine and Chemokine Measurement

The concentrations of inflammatory cytokines, including IL-6, TNF-α, IL-1β, and IL-17A, were quantified in the culture supernatants utilising enzyme-linked immunosorbent assay kits according to the manufacturer’s protocol (Invitrogen, Carlsbad, CA).

### Statistical Analysis

All statistical data were analysed using GraphPad Prism v10. A comparison of mean age between patients and controls was performed by Student’s t-test. The mean cytokine levels among patients and healthy controls in different types of stimulations were compared by oneway analysis of variance (ANOVA) followed by Tukey’s post hoc test, which compares the means of one column with those of every other column. P-values less than 0.05 were considered statistically significant.

## RESULTS

### Patient characteristics

The study cohort comprised 41 female patients diagnosed with systemic lupus erythematosus (SLE) and 36 age-matched healthy female controls, with mean ages of 27.65 and 28.01 years, respectively. The SLE patients exhibited an average disease duration of 2.83 years and a mean age at disease onset of approximately 26.46 years. The mean SLE disease activity score was 10.26, indicating moderate disease activity within the group. Common clinical manifestations among the SLE cohort included oral ulcers (58%), nephritis (54%), arthritis (52%), and malar rash (43%), while less frequent presentations were carditis (14%), autoimmune haemolytic anaemia (8%), and pneumonitis (8%). All patients were receiving standard treatments, including prednisolone and hydroxychloroquine, with a minority also receiving azathioprine or mycophenolate mofetil.

### Titration of bromelain concentration for the stimulation study

**Figure 1.**
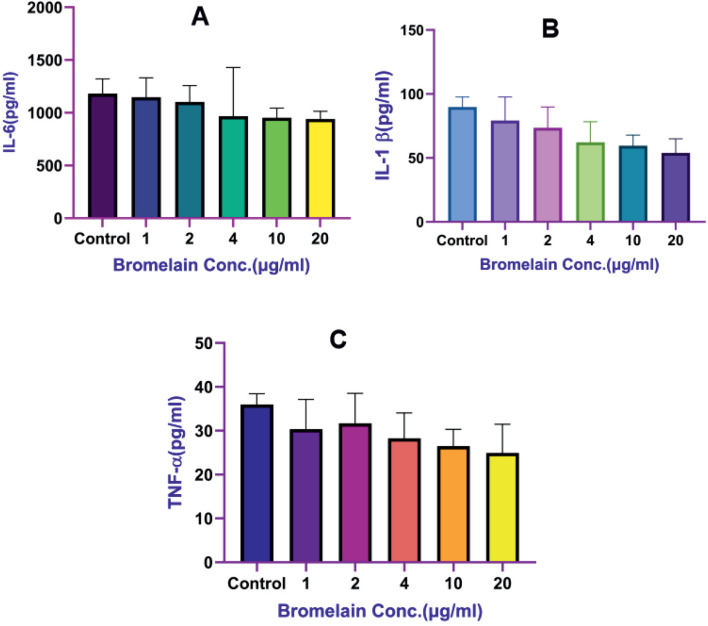
Titration of an appropriate bromelain dose for the in vitro stimulation study. PBMCs were isolated from healthy subjects (n=5). The cells were then stimulated with different doses of bromelain (1ug/mL, 2ug/mL, 4ug/mL, 10ug/mL, and 20 ug/mL) and cultured for 12 hours in a CO2 incubator. IL-1b, TNF-α, and IL-6 levels were quantified in the supernatant using ELISA.

To determine the optimal concentration of bromelain for the in vitro stimulation study, cultured PBMCs were exposed to varying concentrations of bromelain: 1 μg/mL, 2 μg/mL, 4 μg/mL, 10 μg/mL, and 20 μg/mL. In addition to these differential doses of bromelain, a control without bromelain was included. After 12 hours of stimulation, the supernatants were analysed for IL1b, TNF-α, and IL-6 levels. As illustrated in **Figure 1**, the controls exhibited higher levels of IL1b, TNF-α, and IL-6 compared to the wells stimulated with bromelain. A dose-dependent decrease in IL1b, TNF-α, and IL-6 levels was observed with increasing concentrations of bromelain. The diminishing trends for all cytokines continued up to the dose of 10 μg/mL, with cytokine levels remaining similar at 20 μg/mL bromelain, suggesting that 10 μg/mL of bromelain stimulation would be appropriate for the in vitro study of bromelain. Furthermore, a previous study also utilised 10 μg/mL of bromelain for investigating the anti-inflammatory role of bromelain.

### Cell death rate after stimulation with bromelain and lipopolysaccharide

**Figure 2.**
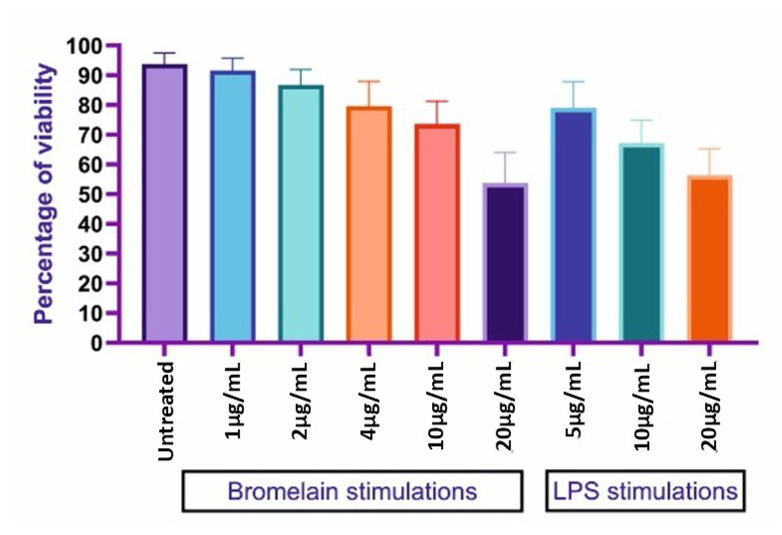
Effect of bromelain and lipopolysaccharide stimulation on cell death in a controlled cell culture method. Human PMBCs were stimulated with varying concentrations of bromelain and lipopolysaccharide for 12 hours. Adherent cells were examined for viability percentage using trypan blue exclusion staining. Data are presented as mean ± SD for five independent experiments.

The data presented in **Figure 2** illustrates that initially, a high percentage of cells remained viable (93.8%) in the absence of stimulation. However, as the dose of bromelain increased, there was a corresponding increase in cell death, with viability dropping to 73.6% at 10 μg/mL and sharply declining to 53.8% at 20 μg/mL. This suggests that 10 μg/mL is the optimal dose for the study. Similarly, with LPS, 5 μg/mL resulted in 79% cell viability, while 10 μg/mL and 20 μg/mL resulted in 67.2% and 56.4% viability, respectively, indicating that 10 μg/mL would be suitable for the stimulation study with LPS as the stimulant.

### In vitro study reveals bromelain is anti-inflammatory

**Figure 3.**
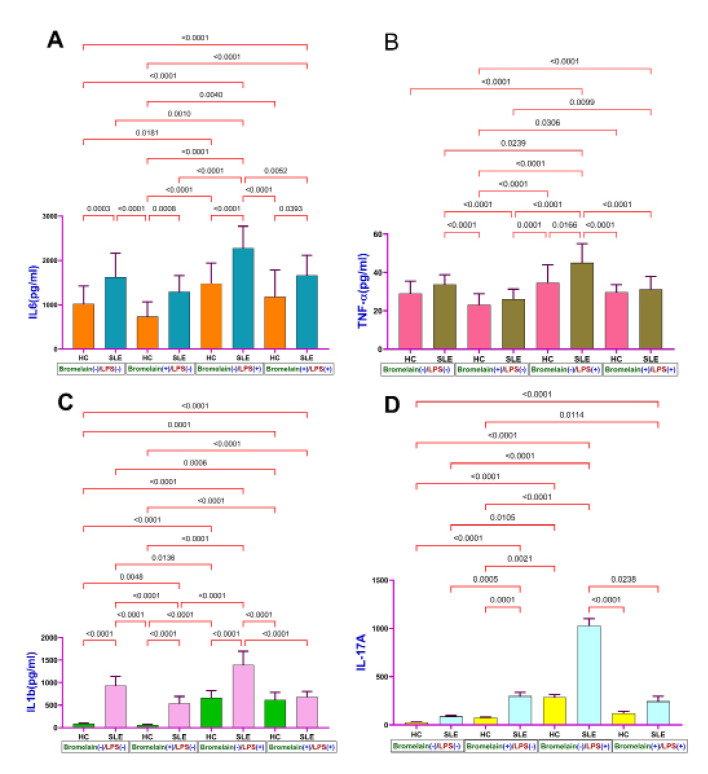
Differential production of inflammatory cytokines with stimulation of PBMCs of SLE patients and healthy controls with bromelain and lipopolysaccharide. Peripheral blood mononuclear cells (PBMCs) from patients with systemic lupus erythematosus (SLE) (n=41) and healthy controls (n=36) were stimulated with various combinations of 10 μg/mL bromelain and/or 10 μg/mL lipopolysaccharide for 12 hours. After that, the supernatants were harvested and quantified for IL-6 (A), TNF-α (B), IL-1β (C), and IL-17A (D) levels using the enzyme-linked immunosorbent assay (ELISA). The mean cytokine levels were compared among the different categories using analysis of variance (ANOVA) followed by Tukeys post-hoc comparison. A p-value less than 0.05 was considered statistically significant.

PBMCs from both SLE patients and healthy controls were cultured and then stimulated using four different combinations of bromelain and lipopolysaccharide: a) without bromelain and LPS as controls, b) with bromelain but without LPS, c) without bromelain but with LPS, and d) with both bromelain and without LPS. After 12 hours, the cultured supernatant was collected and analysed for levels of IL-6, TNF-α, IL-1β, and IL-17A. Levels of the cytokines are shown in **Figure 3**.

In the unstimulated cultured cells, patients with SLE exhibited significantly higher levels of IL-6 (P=0.0003) and IL-1β (P<0.0001) than healthy controls (**Figures 3A** and **C**). A similar trend was observed for two other cytokines, IL-17A and TNF-α; however, the differences did not reach statistical significance (**Figures 3B** and **D**).

Regardless of stimulation combinations, the IL-6 levels in the cultured supernatants were higher in the SLE patients compared to healthy controls. Although not statistically significant, the cells stimulated with bromelain exhibited lower levels of IL-6 in both patients and controls than those without any stimulation, suggesting that bromelain reduced the production of IL-6. Moreover, cells stimulated with only LPS demonstrated higher levels of IL-6 in both SLE patients and healthy controls compared to the other two combinations, namely bromelain (+)/LPS (−) (SLE patients: P<0.0001, HC: P<0.0001) and bromelain (+)/LPS(+) (SLE patients: P=0.005), indicating an anti-inflammatory role of bromelain through the reduction of IL-6 levels. The same trend was observed for other inflammatory cytokines, including TNF-α (**Figure 3B**), IL-1β (**Figure 3C**), and IL-17A (**Figure 3D**). Cells stimulated with bromelain showed lower cytokine levels than those stimulated with LPS. Notably, both control and SLE cells treated with bromelain and LPS consistently displayed lower cytokine levels than those stimulated with LPS alone (IL-6: P=0.005, TNF-α: P<0.0001, IL-1β: P<0.0001, IL-17A: P=0.02), further supporting the anti-inflammatory effect of bromelain in SLE.

## DISCUSSION

The present *in-vitro* study provides evidence of the anti-inflammatory effects of bromelain. In healthy controls and SLE patients, bromelain significantly reduced the production of inflammatory molecules such as TNF-α, IL-1β, IL-17A, and IL-6. Notably, bromelain demonstrated the ability to minimise the production of inflammatory cytokines when stimulated with LPS, highlighting its potential use in controlling cytokine storms in gram-negative infections. The observed reduction in the production of pro-inflammatory mediators suggests that bromelain may hold therapeutic potential in managing SLE.

Corroborating with the earlier reports from our group,^4,26–28^ majority of patients in the enrolled cohort had lupus nephritis. In addition, other clinical phenotypes such as arthritis, malar rash, and oral ulcers were more prevalent. These findings further support the clinical presentation of SLE patients in the eastern Indian cohort.

The dosage of a drug is dependent on the bioavailability of the molecules in the bloodstream. A previous study demonstrated that oral administration of bromelain at a dose of 3 grams/day increased plasma concentration up to 5000 picograms per mL by 48 hours, with an average of 10.8 micrograms/mL of bromelain present in the plasma for 3 to 51 hours.^29^ Additionally, the half-life of bromelain in the plasma has been reported to be 6 to 9 hours.^29^ Based on these observations and the minimal cell death rate at the dose of 10 μg/mL revealed from the current study, we investigated the anti-inflammatory effect of bromelain using 10 μg/mL. We observed that bromelain effectively suppresses inflammatory molecules in PBMCs obtained from SLE patients and healthy controls. Intriguingly, when combined with LPS, bromelain also significantly decreases the production of inflammatory molecules. Similar findings have been reported previously in Raw 264.7 cell lines, where pre-exposure to bromelain reduced LPS-induced inflammation by inhibiting the NF-κB and MAPK signalling pathways.^30^ Additionally, bromelain hinders the migration of neutrophils to different sites by explicitly targeting the CD128 chemokine receptor.^31^ Neutrophils interact with various immune cells and are believed to play a significant role in the induction of autoantibodies. In contrast to our findings, studies have shown that bromelain can stimulate the production of inflammatory modulators such as IL-6, IL-1β, and TNF-α in mouse macrophages and human PBMCs.^32,33^ However, it has been noted that the effects of bromelain depend on the cellular environment. In inflamed or primed inflammatory conditions, bromelain suppresses the production of proinflammatory molecules, as observed in inflammatory bowel disease.^34^ SLE patients displayed higher levels of inflammatory molecules. Bromelain administration reduces inflammation levels and could be promising for SLE management. Although the present study successfully demonstrated the anti-inflammatory role of bromelain, several limitations must be addressed. First, the investigation is an in vitro study; exploration using an in vivo model would be ideal. Second, the mechanism by which bromelain suppresses the production of inflammatory molecules has yet to be identified. Third, the total number of samples considered for the present report is relatively small. Future studies should investigate the effects of bromelain in animal models of SLE and evaluate its efficacy in clinical trials involving SLE patients.

## CONCLUSION

The *in-vitro* study highlights bromelain’s potential anti-inflammatory effects in the context of SLE. The findings suggest that bromelain could serve as a promising therapeutic agent for managing SLE by modulating the dysregulated immune response and mitigating the chronic inflammatory processes associated with the disease.

## Data Availability

Data will be made available on request.
